# Artenimol–piperaquine in children with uncomplicated imported falciparum malaria: experience from a prospective cohort

**DOI:** 10.1186/s12936-019-3047-9

**Published:** 2019-12-16

**Authors:** Lauren Pull, Jean-Marc Lupoglazoff, Matthew Beardmore, Jean-François Michel, Pierre Buffet, Olivier Bouchaud, Jean-Yves Siriez

**Affiliations:** 1 Service D’Accueil Des Urgences Pédiatriques, Hôpital Robert Debré, Assistance Publique-Hôpitaux de Paris, 48 Boulevard Sérurier, 75019 Paris, France; 2 Manchester Royal Infirmary, Manchester, UK; 3 Hôpital Necker-Enfants Malades, Assistance Publique-Hôpitaux de Paris, 75015 Paris, France; 4 Hôpital Avicenne, Assistance Publique-Hôpitaux de Paris, Université Paris 13, 93000 Bobigny, France

**Keywords:** Imported malaria, Children, Artenimol–piperaquine, QTc interval

## Abstract

**Background:**

Although malaria remains one of the major public health threats in inter-tropical areas, there is limited understanding of imported malaria in children by paediatricians and emergency practitioners in non-endemic countries, often resulting in misdiagnosis and inadequate treatment. Moreover, classical treatments (atovaquone-proguanil, quinine, mefloquine) are limited either by lengthy treatment courses or by side effects. Since 2010, the World Health Organization (WHO) has recommended the use of oral artemisinin-based combination therapy for the treatment of uncomplicated *Plasmodium falciparum* malaria worldwide. The benefits of artenimol–piperaquine in children have been validated in endemic countries but experience remains limited in cases of imported malaria.

**Methods:**

This prospective observational study in routine paediatric care took place at the Emergency Department, Robert-Debré Hospital (Paris, France) from September 2012 to December 2014. Tolerance and efficacy of artenimol–piperaquine in children presenting with the following inclusion criteria were assessed: *P. falciparum* positive on thin or thick blood smear; and the absence of WHO-defined features of severity.

**Results:**

Among 83 children included in this study, treatment with artenimol–piperaquine was successful in 82 children (98.8%). None of the adverse events were severe and all were considered mild with no significant clinical impact. This also applied to cardiological adverse events despite a significant increase of the mean post-treatment QTc interval.

**Conclusion:**

Artenimol–piperaquine displays a satisfying efficacy and tolerance profile as a first-line treatment for children with imported uncomplicated falciparum malaria and only necessitates three once-daily oral intakes of the medication. Comparative studies versus artemether-lumefantrine or atovaquone-proguanil would be useful to confirm the results of this study.

## Background

Malaria is a febrile illness caused by the protozoan parasite *Plasmodium* species, transmitted to humans by the bite of infected female *Anopheles* mosquitoes. The main species known to infect humans are *Plasmodium falciparum*, which causes the majority of severe cases, *Plasmodium vivax*, *Plasmodium malaria, Plasmodium ovale* and *Plasmodium knowlesi*. Malaria remains one of the major public health threats in inter-tropical areas. Although the incidence of malaria has fallen since 2010, there is no significant progress in reducing malaria cases for the period 2015–2017 [1].

Resistance to artemisinin is a major challenge and vaccine development is still far from providing long-lasting benefits [2]. In the early 21st century, artemisinin-based combinations were introduced to Africa in response to increasing *P. falciparum* resistance to conventional anti-malarial drugs and to improve treatment efficacy. Qinghao (*Artemisia annua*) has been used by Chinese botanists for many centuries for the treatment of fever and the active component of the plant (qinghaosu or artemisinin) was purified in China in 1972 [3]. Since 2010, the World Health Organization (WHO) has recommended the use of oral artemisinin-based combination therapy (ACT) for the treatment of uncomplicated *P. falciparum* malaria worldwide [4]. With 5220 estimated cases in 2017 (313 declared in children), France is the developed country most affected by imported malaria [5]. Imported malaria in children is not well recognized by paediatricians and emergency practitioners, resulting in misdiagnosis and inadequate treatment. On the other hand, classical treatments (atovaquone-proguanil, mefloquine, quinine) can be limited by their lengthy treatment courses and potential side effects (vomiting, neuropsychiatric adverse effects, cinchonism) [6, 7]. Artenimol–piperaquine (AP) was approved for clinical use in adult and paediatric patients in France in 2012. While the safety and efficacy of AP in children has been validated in endemic countries [8, 9], the experience with this drug is still very limited in imported malaria, with very few publications in adults [10] and none in children. Interestingly, two cases of AP treatment failure in imported uncomplicated falciparum malaria have been recently reported in adults [11]. The results of a 3 year experience of treating children with uncomplicated, imported falciparum malaria (UFM) with AP are reported in this article.

## Methods

This prospective observational cohort study in routine paediatric care took place in the Emergency Department, Robert-Debré Hospital (Paris, France) from September 2012 to December 2014. All children presenting with a fever, or recent history of fever, and returning from a malaria endemic country within the last 3 months were screened for malaria (thin and thick blood smear). UFM was defined by fever or a recent history of fever, a *P. falciparum* positive thin or thick blood smear and the absence of World Health Organization (WHO)-defined features of severity. Parasitaemia count > 4%, when it was an isolated finding, was not considered a criterion of severity, as suggested by the 2007 French recommendations [12]. According to local guidelines, AP is the first-line option treatment recommended, but practitioners-on-duty are free to prescribe one of the three anti-malarials available in France (atovaquone–proguanil, artemether–lumefantrine or AP). All children presenting with UFM and treated with AP were included in the study.

Severe malaria or non-falciparum malaria patients were not included in the study and were treated with intravenous artesunate or oral chloroquine, respectively. Patients treated with another drug than AP were not included in the study. Following the manufacturer’s recommendations, children received the 40 mg/320 mg AP tablets (the only dosage available in France) according to body-weight (7 ≤ 13 kg: ½ tablet; 13 ≤ 24 kg: 1 tablet; 24 ≤ 36 kg: 2 tablets; 36 ≤ 75 kg: 3 tablets; 75–100 kg: 4 tablets) on an empty stomach. When children were unable to swallow tablets, they were crushed and then given in very sweet yoghurt. In one case, nasogastric tube was used for drug administration. If the child vomited less than half an hour or between half an hour and 1 h after ingestion, a complete dose or half of the dose was re-administered, respectively. Nurses observed the child for 1 h and, in the absence of vomiting, significant asthenia or any symptoms or reason (including parental inability to care for child at home) requiring hospitalization, the child was discharged home. At the time of hospital discharge, the parents were given the rest of the treatment free-of-charge in order to administer it to their child at home for 2 days, every 24 h, on an empty stomach.

A 12-lead electrocardiogram (EKG) was performed before starting AP and at the first follow-up visit. The QTc interval was calculated by an experienced cardio-rhythmologist. A paired T*-*test was performed to determine whether there was a statistically significant difference in mean QTc before and after starting treatment with AP. Post-treatment clinical and laboratory follow-up (including thin and thick smear) were undertaken first between day 3 and day 8 and on a second occasion on day 28. Parents were informed of the study and gave their consent. The study protocol was approved by the Robert-Debré Hospital (Assistance Publique-Hôpitaux de Paris) ethical committee.

## Results

**Fig. 1:**
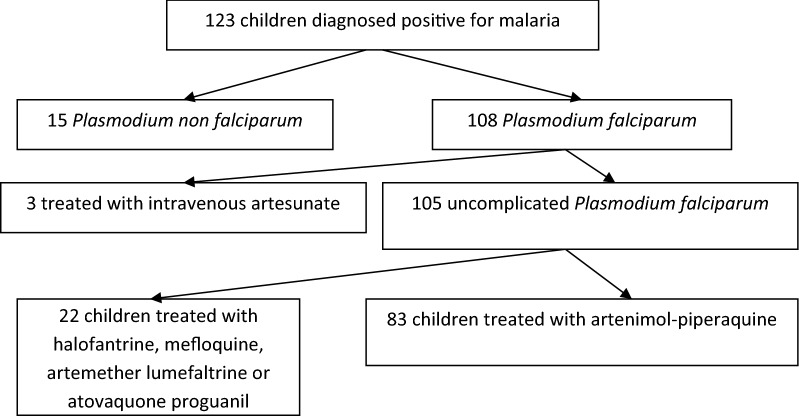
Flow chart of children presenting with malaria at Robert-Debré hospital between September 1 2012 and December 31 2014

During the study period, malaria was diagnosed in 123 children, 3 of them had severe malaria and 15 had non-falciparum malaria. 22 children with UFM were treated with drugs other than AP and were not included in the study (Fig. 1). In total 83 children, 39 girls and 44 boys (sex ratio 1.13), met the inclusion criteria and were considered in the final analysis. Mean age was 9.6 years (13 months–16.8 years). The study population median weight was 34,5 kg with an interquartile range (IQR) of [22,25–48 kg]. All patients were of sub-Sahara African origin and had visited their families in their country of origin, mainly Ivory Coast (42%) and Mali (29%). On admission, median parasitaemia was 1% with an interquartile range (IQR) of 0.4% to 2.6%. The majority (70/83; 84.3%) returned home 1 hour after taking their first dose of treatment. All patients had negative thin and thick blood smears between day 3 and day 8 after the beginning of treatment. The cure-rate was 98.8% as 82/83 children were confirmed malaria-negative between day 22 and day 56 (mean D 31.6). One child had a recrudescence on day 23. Retrospectively, his mother subsequently stated that he had vomited the two doses given at day 2 and day 3 at home, though this was not declared at the day 3 follow-up visit, at which the child’s parasitaemia count was negative. The child received three additional doses of AP via nasogastric tube at the hospital and the subsequent microscopic examinations both 3 and 30 days later were negative. One child originating from Ivory Coast returned to his country before the day 28 control. During a telephone conversation 2 months following treatment, formal assurance was provided by the parents that the child had been clinically well without febrile episode.

Mean haemoglobin level was 10.74 g/dL (confidence interval 10.31–11.16; range 5–15.5) and 11.67 g/dL (confidence interval 10.3–13.1) on admission and on D30, respectively. Nine children (10.84%) vomited after one of the doses; all of them repeated AP according to the protocol without vomiting again. It is not possible to determine whether vomiting was a drug adverse effect or malaria symptom, or a combination of both.

Evolution of QTc intervals was analysed in children who had EKG both before and after treatment (72.3%). EKG post treatment was performed within 24 h of the end of the treatment in 80% of the cases; 20% were registered between 24 and 72 h after the end of the treatment. The average pre-treatment QTc was 393 ms ± 2 s.d. whereas the average post-treatment QTc was 405 ms ± 2 s.d. (p < 0.01), children being afebrile after treatment. Before treatment, one febrile child had a QTc at 447 ms but post-treatment QTc was normal at 435 ms with no fever. Six patients with a normal QTc pre-treatment (less than 440 ms), had a prolongation of the QTc to 450 ms in 5 cases and to 490 ms in one case, without symptoms.

It should be noted that none of the 83 included children in this study were taking concomitant medication other than paracetamol and, therefore, the risk of interaction with artenimol piperaquine could be ruled out.

## Discussion

In this prospective observational study in routine paediatric care in France, one course of AP was successful in 82/83 children (98.79%) with uncomplicated *P. falciparum* malaria. One child had a recrudescence probably related to immediate vomiting of two doses given at home and was subsequently cured after receiving three additional doses of AP. In conclusion, AP reached the WHO-recommended efficacy (> 95%) in this population [4].

Adverse events were rare and in no children was it deemed necessary to switch to another anti-malarial drug. The aryl-amino-alcohol compounds, of which piperaquine is a member, can prolong the QTc interval. The inhibitory concentration 50 (IC50), an indirect measure of this risk, is the average serum concentration of the molecule required to block 50% of potassium channels during phase 3 repolarization of cardiac cells. The IC50 of piperaquine (0.11 µmol/L) is six times greater than that of halofantrine (0.018 µmol/L), known to demonstrate cardiotoxicity, making it less likely to induce marked, life-threatening prolongation of the QT interval. It is, however, lower than that of chloroquine (1 µmol/L) or lumefantrine (2.6 µmol/L) [13], which is the partner drug of artemether. The findings of this study are in line with other studies performed in children in endemic zones, by showing an occasional and moderate prolongation of QTc without cardiac-related symptoms [14–16]. All the children, except one, were examined between day 22 and day 56. No child demonstrated any clinically detectable cardiac problem. One child returned home to Africa before the D28 control but formal assurance was provided by the parents that the child had been clinically well 2 months following treatment. There is still controversy with regards to acceptable treatment-induced QTc prolongation. Reassuringly however, in a recent meta-analysis on children receiving intermittent preventive treatment for malaria with AP, Gutman et al. [17] reported that serious adverse events were less frequent with AP than with other drugs or placebo; and that there was no significant supplementary increase in QTc prolongation with increased courses of AP

In this study, moderate anaemia was infrequent, and severe anaemia was rare (8/83 and 1/83 patients, respectively), possibly due the predominance of older children (95.2%) over children < 24 months. Furthermore, despite a follow-up period lasting 22 to 56 days after treatment initiation, no episodes of severe haemolytic anaemia were reported unlike that which has been recently reported after treatment of severe malaria with intravenous artesunate [18] or with oral artemisinin combination [19]. This was probably related to the low prevalence of hyperparasitaemia in this cohort. While current practice in Northern countries recommends that children with UFM should be hospitalized for initial treatment, 84.3% of the children in this study returned home 1 hour after taking their first dose of treatment with favourable clinical evolution apart from the single uncomplicated relapse reported above [12, 20, 21]. A recent review on imported paediatric malaria wisely suggests that the decision to manage a child with UFM without hospital admission should be made by experienced clinicians in children generally over 5 years of age, presenting with low parasitaemia and for whom reliable follow-up is possible [22]. However, the results of this study suggest that it is also possible to treat children under the age of 5 and with parasitaemia up to 10% as outpatients provided that the parents are informed, considered, reliable and that the scheduled follow-up is adhered to strictly.

The D3, D7 and D28 WHO-recommended follow-up visits are difficult to implement in this particular patient group. This seems mainly due to the fact that, even when parents are considered reliable, almost all patients display complex family dynamics resulting in missed or delayed appointments. Follow-up phone calls are often necessary to rearrange visits. Nevertheless, we have been able to follow up all our children with two controls, between day 3 and day 8 and between day 22 and day 56, respectively. Only one child had no second control; his parents informed us by phone from Ivory Coast—where the family lived—that the child was clinically well 2 months after the end of the treatment. The overall follow-up rate at least 1 month after treatment was 98%.

There are some limitations to this study. The first is a lack of comparison treatment arm of the study. In France, two artemisinin-based combinations are recommended as treatment for UFM, artemether–lumefantrine (AL) and AP. 30 children with AL were treated in 2011–2013; all children were cured, and adverse events were rare. However, the marked difference in the number of cases in each group rendered it inappropriate to compare the two molecules. A randomized study comparing AL and AP would have been difficult to perform, especially in paediatric practice. Additionally, comparative randomized studies often imply selection bias. This is the reason why a cohort study design was chosen to assess the efficacy and safety of AP in children in real-life conditions. Based on years of experience acquired at the Robert Debré Emergency Department, it appears that AP displays two advantages over AL. First, only one dose is required daily, instead of two doses for AL, which sometimes obliges parents to wake the child during the night. Secondly, and more importantly, it is recommended to give AP on an empty stomach while AL must be taken with food to improve lumefantrine absorption, which is a limitation given that food ingestion in children with acute malaria may induce vomiting. A second potential limitation of this study is the timing of the second EKG recording, between day 3 and day 8 (period of the first scheduled follow-up visit) compared to day 2 in the majority of other studies. It could be assumed that due to the long half-life of piperaquine, the impact of this delay in QTc prolongation is probably not clinically significant.

## Conclusion

Imported malaria in children is a rare illness, whose initial management by both paediatricians and general practitioners is frequently sub-optimal, necessitating both a simple and well-tolerated treatment. Artemisinin-based oral combinations have markedly improved the treatment of malaria for children who live in or travel to endemic countries and are nowadays the reference first-line drugs. Artenimol–piperaquine has the advantage of being taken without food and with a simple dosing schedule. Moreover, this study did not show any cardiotoxicity.

Based on the high observed cure-rate and good safety profile, DP appears as a valuable first-line option to treat children in the context of imported malaria.

## Data Availability

The datasets used and/or analysed during the current study are available from the corresponding author on reasonable request.

## Abbreviations

WHO: World Health Organization
ACT: artemisinin-based combination therapy
AP: artenimol–piperaquine
UFM: uncomplicated imported falciparum malaria
EKG: electrocardiogram
IQR: interquartile range
IC50: inhibitory concentration 50
AL: artemether–lumefantrine
